# A Rare Case of Uterine-Origin Vulvar Leiomyoma Occurring in a Juvenile Girl: Case Report and Literature Review

**DOI:** 10.1155/crog/8874924

**Published:** 2025-04-27

**Authors:** Emiko Hamada, Naomi Shiga, Mika Watanabe, Satoko Sato, Zen Watanabe, Masahito Tachibana, Masatoshi Saito

**Affiliations:** ^1^Department of Obstetrics and Gynecology, Sendai Medical Center, Sendai City, Miyagi Prefecture, Japan; ^2^Department of Obstetrics and Gynecology, Suzuki Memorial Hospital, Iwanuma City, Miyagi Prefecture, Japan; ^3^Department of Diagnostic Pathology, Tohoku Kosai Hospital, Sendai City, Miyagi Prefecture, Japan; ^4^Department of Diagnostic Pathology, Tohoku University Hospital, Sendai City, Miyagi Prefecture, Japan; ^5^Department of Obstetrics and Gynecology, Tohoku University Hospital, Sendai City, Miyagi Prefecture, Japan

**Keywords:** juvenile girl, leiomyoma, mesenchymal tumor, vulva

## Abstract

Leiomyoma arising from the vulva is very rare, and it is difficult to differentiate it from a Bartholin gland cyst and aggressive angiomyxomas. We report a case of vulvar leiomyoma in a juvenile girl. The patient, a 16-year-old female, had noted a tender subcutaneous nodule on the right vulva for 2 years. At first, a Bartholin gland cyst was suspected, and she was prescribed antibiotics. However, because of persistent symptoms, the patient was referred to our clinic for further examination. An MRI scan suspected a mesenchymal tumor, and surgical resection was performed. Tumor cells were diffusely positive for anti-smooth muscle antibodies, HHF35, and Desmin, and leiomyoma was suspected. Immunostaining tests were negative for estrogen receptor and positive for progesterone receptor. The patient exhibited an excellent postoperative course with no evidence of recurrence at the latest follow-up. Surgical resection is the only curative treatment, and long-term follow-up is recommended because of rare reports of recurrence.

## 1. Introduction

Uterine leiomyoma is a common disease and is frequently treated in daily medical practice. On the other hand, leiomyoma occurring in the vulva (hereafter referred to as vulvar leiomyoma) is very rare, accounting for approximately 0.03% [1] of all gynecological tumors. They are often misdiagnosed as Bartholin gland cysts, and other differentials include aggressive angiomyxoma (AAM), angiomyofibroblastoma (AMFB), and fibromas. We report a case of a juvenile female with a painful vulvar mass that was difficult to diagnose. Despite preoperative suspicions of Bartholin gland cyst or mesenchymal tumors, histopathological examination following surgical excision confirmed the presence of vulvar leiomyoma.

I hope that this report can contribute to one of the differential diagnoses in future diagnoses.

This report received approval from the ethics committee of Tohoku University School of Medicine (Review No: Receipt-36002), and the patient's informed consent was duly obtained.

## 2. Case Presentation

The patient, a 16-year-old female, presented with a complaint of a painful vulvar mass. Her medical and family history did not reveal any notable information, and there were no records of specific medication or allergy history. Additionally, she had no history of pregnancy, delivery, or sexual intercourse. Menarche occurred at the age of 13, with a regular menstrual cycle and no reported dysmenorrhea.

For a duration of 2 years, she had experienced tenderness associated with a 10 mm subcutaneous nodule on the right vulva. Consulting a local gynecologist, the initial suspicion was a Bartholin's gland cyst, leading to antibiotic treatment. However, with no improvement, she remained under follow-up care. In the subsequent year, as vulvar pain persisted, she was referred to another gynecology department. Ultrasonography showed blood flow inside the mass, so a Bartholin gland cyst was ruled out. The patient was referred to our hospital for a thorough examination and treatment of the vulvar mass.

**Figure 1:**
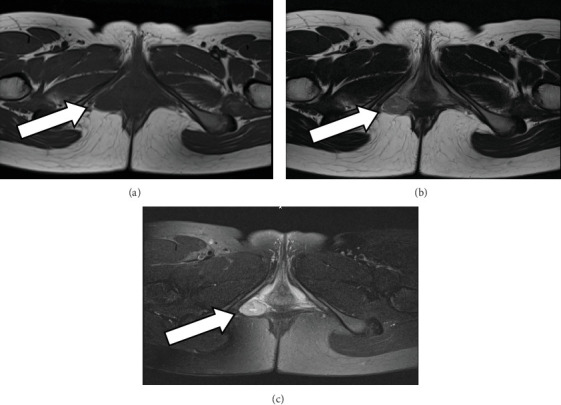
Imaging findings. Magnetic resonance imaging revealed a mass extending between the muscles of the transverse perineal muscle, the sciaticus spongiosus muscle, and the saphenofemoral muscle. (a) T1-weighted images and (b) T2-weighted images displayed low signal intensity, while (c) fat-suppressed T2-weighted images exhibited high signal intensity, indicative of a mesenchymal tumor.

Upon initial examination, a palpable mass measuring 30 mm in size was identified on the right vulva, exhibiting good mobility and tenderness on palpation. MRI scan (Figure 1) revealed a bifid, bicameral, internal, substantial mass, located laterally in contact with the right lateral posterior vaginal wall and cephalad in contact with the anorectal erector spinae muscle and entering between the muscles of the transverse perineal muscle, the ischiocavernous muscle, and the globus pallidus muscle. T1-weighted and T2-weighted images showed low signal intensity, and fat-suppressed T2-weighted images showed high signal intensity, suggestive of a mesenchymal tumor such as AAM or AMFB.

Transcutaneous excision of subcutaneous tumor was conducted under general anesthesia. The identified mass was found in proximity to the bulbospongiosus and ischiocavernous muscle, with its cephalic side in contact with the anorectal elevator muscle. Enveloped by a capsule exhibiting a smooth surface, the mass proved easily detachable from the surrounding area. The operative time, the recorded bleeding, and the specimen weight totaled 64 min, 8 g, and 7 g, respectively. The postoperative course was favorable, leading to the patient's discharge on the third postoperative day. No recurrence was observed during the postoperative follow-up.

**Figure 2:**
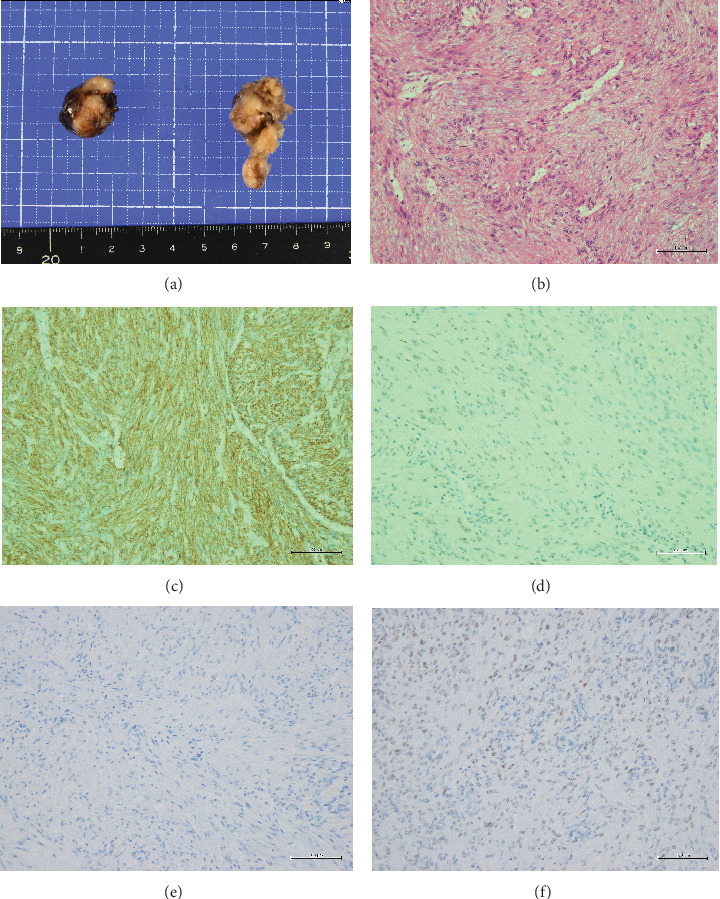
Macroscopic and pathological findings. (a) Macroscopic view of the resected specimen. (b) Hematoxylin and eosin staining (×200) showed that spindle-shaped tumor cells with mildly enlarged nuclei and eosinophilic cytoplasm are proliferating in bundles, intertwining with each other. Muscular markers such as (c) SMA (×200) and (d) HHF (×200) were diffusely positive. (e) Estrogen receptor (×200) was negative, and (f) progesterone receptor (×200) was positive.

Histopathological findings (Figure 2) revealed spindle-shaped cells with mildly enlarged nuclei and eosinophilic cytoplasm, displaying intermingled proliferation with mild edematous changes. Tumor cells were diffusely positive for the muscle markers anti-smooth muscle antibody (SMA) and HHF35, confirming the diagnosis of leiomyoma. Estrogen receptors (ERs) were negative, and progesterone receptors (PRs) were positive.

## 3. Discussion

Leiomyomas can manifest in various locations, including smooth muscle cells. While their predominant occurrence is within the myometrium of the uterus, leiomyomas have been documented in other anatomical sites such as the urethra, bladder, and peritoneum [2]. Despite it being reported to occur in a wide range of age groups, the most common age group is in the 30s and 40s, with only a few reports in teenagers [3]. In general, vulvar leiomyomas present as painless, solitary, and localized growths [3], characterized by slowly enlarging. However, if the tumor enlarges and compresses peripheral nerves, symptoms such as pain, erythema, and dyspareunia may occur [1].

Vulvar leiomyoma is commonly misidentified as a Bartholin gland cyst, relying on historical data, symptomatology, and physical examination findings [4, 5]. Other diseases that should be differentiated include AAM, AMFB, fibroma, smooth muscle tumors, and abscesses [2, 3, 6]. Ultrasonography is the most frequently performed imaging test due to its simplicity and cost-effectiveness. MRI is useful in cases of diagnostic difficulty or to determine benign or malignant status. Leiomyoma typically reveals low signal intensity to smooth muscle on T2-weighted images, isointensity on T1-weighted images, and uniform enhancement on contrast-enhanced images [1].

Histopathologic examination stands as the definitive diagnostic approach for leiomyomas. These lesions exhibit three distinct histologic patterns: spindle-shaped, epithelioid, and myxoid, with the spindle-shaped variant being the most prevalent [5, 7]. Tumor cells in leiomyomas assume a spindle-shaped configuration, featuring oval to elongated nuclei and abundant acidophilic cytoplasm. These cells are organized in bundles and intermingled arrangements [7]. Although some cases of AAM may exhibit positivity for smooth muscle markers such as vimentin, desmin, and SMA, the pathophysiologic findings are different. The presence of a positive CD34 marker for hematopoietic stem cells serves as a distinguishing feature [8]. It is essential to recognize that, despite the presence of muscle markers in some AAM cases, the histopathological findings can vary significantly [8]. In this case, a differential diagnosis of AAM was contemplated but ultimately ruled out based on distinct histopathological features, including the absence of the characteristic thick vascular wall seen in AAM, different cellular morphology, and diffuse positivity for myogenic markers.

ER and PR are highly expressed in uterine leiomyoma, and both estrogen and progesterone are thought to promote tumor development [9]. Vulvar leiomyoma has also been suggested to be an estrogen-dependent tumor, based on reports that estrogen/progesterone therapy is associated with recurrence of vulvar leiomyoma [9] and reports of cure using ER modulators for vulvar leiomyoma [10]. On the other hand, vulvar leiomyomas have been reported to arise from smooth muscle cells of erectile tissue, vascular wall, round ligament, and pisiform muscle [11]. They may lack intrinsic ER and PR expression. There are also reports of a significant decrease in ER expression in the epidermis and fibroblasts from the vaginal to labia minora, labia majora, and pubic epithelium [11]. Additionally, ER and PR expressions were not observed in skin appendages and blood vessels in the vulva [11]. Previous reports on vulvar leiomyoma included two cases that were negative for both ER and PR [12, 13], and we could not find ER-negative and PR-positive cases like this case in the previous reports. Considering this case, it is suggested that they may have originated from uterine smooth muscle cells present in gynecological organs such as the round ligament.

Surgical resection is the only curative treatment for vulvar leiomyoma [5]. Gunnlaugur et al. conducted a clinical and pathological evaluation of 25 vulvar smooth muscle tumors [12], with 19 cases available for a follow-up period averaging 5 years. Among these, 10 exhibited pathological findings of leiomyoma, five were diagnosed as atypical leiomyoma, and four as leiomyosarcoma. Throughout the follow-up, three patients (one with leiomyoma and two with atypical leiomyoma) experienced local recurrence, and one patient diagnosed with leiomyosarcoma died of metastasis. Recurrence in the leiomyoma case manifested more than 10 years after the initial onset. Even upon confirmation of the pathological diagnosis of leiomyoma, it is advisable to provide information regarding the potential for recurrence and recommend a sufficiently extended follow-up.

We have experienced a rare case of vulvar leiomyoma in a 16-year-old female. Owing to its nonspecific clinical presentation, it was difficult to differentiate it from Bartholin gland cysts or mesenchymal tumors such as AAM in preoperative diagnosis. Histopathological examination proved instrumental for a definitive diagnosis, emphasizing the necessity of appropriate surgical resection for both diagnostic and therapeutic purposes. When a vulvar mass does not present as a typical Bartholin gland cyst, a proactive approach involving thorough imaging and surgical resection should be contemplated.

## Data Availability

The data that support the findings of this study are available from the corresponding author upon reasonable request.

## References

[1] da Silva Fontinele D. R., Silva L. H. C., Vieira S. C., das Chagas Santos Pinheiro F., de Alencar Nunes G. (2022). Leiomyoma of the Vulva: Case Report. *Journal of Pediatric & Adolescent Gynecology*.

[2] Fasih N., Prasad Shanbhogue A. K., Macdonald D. B. (2008). Leiomyomas Beyond the Uterus: Unusual Locations, Rare Manifestations. *Radiographics*.

[3] Kurdi S., Arafat A. S., Almegbel M., Aladham M. (2016). Leiomyoma of the Vulva: A Diagnostic Challenge Case Report. *Case Reports in Obstetrics and Gynecology*.

[4] Pandey D., Shetty J., Saxena A., Srilatha P. S. (2014). Leiomyoma in Vulva: A Diagnostic Dilemma. *Case Reports in Obstetrics and Gynecology*.

[5] da Silva Tavares K. A., Moscovitz T., Tcherniakovsky M., de Melo Pompei L., Fernandes C. E. (2017). Differential Diagnosis Between Bartholin Cyst and Vulvar Leiomyoma: Case Report. *RBGO Gynecology & Obstetrics*.

[6] Onishi T., Yonei A., Kekuchi T., Tuchida Y., Tominaga K. (2020). Three Cases of Aggressive Angiomyxoma of the External Genitalia. *Journal of Japan Surgical Association*.

[7] Nucci M. R., Fletcher C. D. (2000). Vulvovaginal Soft Tissue Tumours: Update and Review. *Histopathology*.

[8] Zhao T., Liu X., Lu Y. (2015). Myxoid Epithelial Leiomyoma of the Vulva: A Case Report and Literature Review. *Case Reports in Obstetrics and Gynecology*.

[9] Siegle J. C., Cartmell L. (1995). Vulvar Leiomyoma Associated With Estrogen/Progestin Therapy. A Case Report. *The Journal of Reproductive Medicine*.

[10] Darbhamulla A., Waston A. J. S., Benatar B. (2004). Recurrent Vulval Fibroids—An Unusual Indication for Selective Oestrogen Receptor Modulators (SERMs). *Journal of Obstetrics and Gynaecology*.

[11] Hodgins M. B., Spike R. C., Mackie R. M., MacLean A. B. (1998). An Immunohistochemical Study of Androgen, Oestrogen and Progesterone Receptors in the Vulva and Vagina. *British Journal of Obstetrics and Gynaecology*.

[12] Gunnlaugur N., Andrew R., Frederick K., Robert Y., Robert S. (1996). Smooth-Muscle Tumors of the Vulva. *The American Journal of Surgical Pathology*.

[13] Kajiwara H., Yasuda M., Yahata G. (2002). Myxoid Leiomyoma of the Vulva: A Case Report. *Tokai Journal of Experimental and Clinical Medicine*.

